# Patients with urothelial carcinoma have poor renal outcome regardless of whether they receive nephrouretectomy

**DOI:** 10.18632/oncotarget.11223

**Published:** 2016-08-11

**Authors:** Peir-Haur Hung, Hung-Bin Tsai, Kuan-Yu Hung, Chih-Hsin Muo, Mu-Chi Chung, Chao-Hsiang Chang, Chi-Jung Chung

**Affiliations:** ^1^ Department of Internal Medicine, Ditmanson Medical Foundation Chiayi Christian Hospital, Chiayi, Taiwan; ^2^ Department of Applied Life Science and Health, Chia-Nan University of Pharmacy and Science, Tainan, Taiwan; ^3^ Department of Tramatology, National Taiwan University Hospital, Taipei, Taiwan; ^4^ Department of Internal Medicine, National Taiwan University Hospital, Hsin-Chu Branch, Hsin-Chu City, Taiwan; ^5^ Management Office for Health Data, China Medical University and Hospital, Taichung, Taiwan; ^6^ Division of Nephrology, Department of Medicine, Taichung Veterans General Hospital, Taichung, Taiwan; ^7^ Department of Urology, China Medical University and Hospital, Taichung, Taiwan; ^8^ Department of Health Risk Management, College of Public Health, China Medical University, Taichung, Taiwan; ^9^ Department of Medical Research, China Medical University Hospital, Taichung, Taiwan

**Keywords:** chronic kidney disease, end stage renal disease, nephrouretectomy, urothelial carcinoma

## Abstract

The association between urothelial carcinoma (UC) and subsequent ESRD incidence has not been confirmed. This was a population-based study using claims data from the Taiwan National Health Institutes from 1998 to 2010. The study cohort consisted of 26,017 patients with newly diagnosed UC and no history of ESRD, and the comparison cohort consisted of 208,136 matched enrollees without UC. The incidence of ESRD was ascertained through cross-referencing with a registry for catastrophic illnesses. Cox proportional hazard regression analysis was used to estimate the risk of ESRD associated with UC and UC subtype. A total of 979 patients (3.76%) from the UC group and 1,829 (0.88%) from the comparison group developed ESRD. Multivariable analysis indicated that compared with the comparison group, the hazard ratios (HRs) for ESRD were 7.75 (95% confidence interval [CI]: 6.84 to 8.78) and 3.12 (95% CI: 6.84 to 8.78) in the cohort with upper urinary tract UC (UT-UC) and bladder UC (B-UC), respectively. In addition, there were significantly increased risks for ESRD in UC patients receiving and not receiving nephrouretectomies or aristolochic acids (AA). Moreover, the UC patients receiving segmental ureterectomy and ureteral reimplantation had approximately 1.3-fold and 2.4-fold increased risk for ESRD after control for confounders, respectively. Thus, our data indicate that UT-UC and B-UC independently increased the risk for ESRD in patients after considering about nephrouretectomies or aristolochic acids (AA). In addition, UC patients receiving segmental ureterectomy and ureteral reimplantation had increased risk for ESRD.

## INTRODUCTION

End-stage renal disease (ESRD) is a significant public health issue in Taiwan and internationally because patients require significant healthcare services and suffer from reduced quality of life. Taiwan has had the greatest incidence and the second greatest prevalence of ESRD since 2000 in an international comparison based on data from the United States Renal Data System Annual Data Report [1]. Moreover, during the past 10 years, Taiwan has also confronted a serious challenge because the incidence and prevalence of ESRD have increased 2.6 and 3.7 times, respectively [2]. Substantial epidemiological and clinical evidence indicates links between hypertension, diabetes, obesity, and metabolic syndrome with the onset and/or progression of chronic kidney disease (CKD) [3], although the effect of urinary tract malignancy on CKD is uncertain.

In Taiwan, urothelial carcinoma (UC) is common among patients with CKD and ESRD [4]. Some research suggests that patients with CKD or ESRD have a greater risk for cancer [5, 6]. In particular, the standardized incidence ratio of UC for patients receiving maintenance dialysis was 48.2 compared with the general population [7]. Furthermore, different dialysis modalities and gender may modify the risk for cancer in patients with ESRD [8]. Meanwhile, UC itself may promote the development of ESRD, especially in patients with upper urinary tract-UC (UT-UC) [9]. However, to date, only limited studies have analyzed the effect of UC on the morbidity and mortality of patients with ESRD [4, 9–11].

Thus, we need more evidence to determine the relationship between UC and development and progression of ESRD. In addition, limited research has evaluated the correlation between the location (or subtype) of UC and the incidence of subsequent ESRD. We propose that patients with UC may face greater risk of ESRD and that different UC subtypes are associated with different risks. We used the nationally representative database of the universal insurance program of Taiwan to determine the association between UC and ESRD.

## RESULTS

### Baseline characteristics of study subjects

Table 1 shows the sociodemographic characteristics and comorbidities of the comparison cohort, UC cohort, and cohorts with different UC subtypes. The age and sex of comparison and UC cohort were similar because of matching. There were significant differences in the duration from UC to ESRD between comparison and UC cohort, with the UT-UC cohort having the shortest duration (2.9 years) and the comparison cohort having the longest duration (4.8 years). Comparison of comorbidities in the UC and comparison cohorts indicated that the UC cohort had greater prevalences of hypertension, diabetes, coronary heart disease, atrial fibrillation, heart failure, CKD, and hyperlipidemia (*p* < 0.01 for all comparisons). Comparison of the B-UC and UT-UC cohorts indicated that the UT-UC cohort had higher prevalences of nephrouretectomy and solitary kidney.

**Table 1:** Demographic characteristics and comorbidities of the control, UC, UT-UC, and B-UC cohorts. Except for follow-up years, numbers indicate N (percent)

	Comparison Group	UC	*p ^a^*	UT-UC	B-UC
Total number	208136	26017		4263	21537
Follow-up years (mean ± SD)	4.79 ± 3.43	3.80 ± 3.27	<0.0001	2.94 ± 2.81	3.98 ± 3.33
Age, years			1.0000		
25–44	9496 (4.56)	1187 (4.56)		130 (3.05)	1036 (4.81)
45–54	23688 (11.4)	2961 (11.4)		396 (9.29)	2537 (11.8)
55–64	40504 (19.5)	5063 (19.5)		878 (20.6)	4151 (19.3)
65–74	66096 (31.8)	8262 (31.8)		1596 (37.4)	6591 (30.6)
≥ 75	68352 (32.8)	8544 (32.8)		1263 (29.6)	7222 (33.5)
Mean (SD)	67.5 ± 12.6	68.2 ± 12.4		68.4 ± 11.0	68.2 ± 12.6
Sex			1.0000		
Male	145176 (69.7)	18147 (69.7)		1857 (43.6)	16176 (75.1)
Female	62960 (30.3)	7870 (30.3)		2406 (56.4)	5361 (24.9)
Geographic region			<0.0001		
Northern	86554 (41.6)	8970 (34.5)		861 (20.2)	8047 (37.4)
Central	42928 (20.6)	4888 (18.8)		819 (19.2)	4039 (18.8)
Southern	57490 (27.6)	9601 (36.9)		2139 (50.2)	7360 (34.2)
Eastern	21159 (10.2)	2558 (9.83)		444 (10.4)	2091 (9.71)
Occupation			<0.0001		
White collar	88544 (42.6)	11240 (43.2)		1757 (41.2)	9396 (43.6)
Blue collar	86942 (41.9)	11563 (44.5)		2224 (52.2)	9232 (42.9)
Others	32237 (15.5)	3208 (12.3)		281 (6.59)	2904 (13.5)
Monthly income, NTD			<0.0001		
≤ 15840	118082 (56.7)	14832 (57.0)		2149 (50.4)	12569 (58.4)
15841–20100	9569 (4.60)	2116 (8.13)		387 (9.08)	1700 (7.89)
> 20100	80485 (38.7)	9069 (34.9)		1727 (40.5)	7268 (33.8)
Comorbidities					
Diabetes	18522 (8.90)	4493 (17.3)	<0.0001	900 (21.1)	3557 (16.5)
Hypertension	36179 (17.4)	8635 (33.2)	<0.0001	1753 (41.1)	6808 (31.6)
Coronary heart disease	18544 (8.91)	2988 (11.5)	<0.0001	546 (12.8)	2416 (11.2)
Atrial fibrillation	3796 (1.82)	537 (2.06)	0.0067	83 (1.95)	452 (2.10)
Heart failure	5854 (2.81)	880 (3.38)	<0.0001	168 (3.94)	702 (3.26)
Chronic kidney disease	1266 (0.61)	560 (2.16)	<0.0001	157 (3.68)	400 (1.86)
Hyperlipidemia	8119 (3.90)	1371 (5.27)	<0.0001	280 (6.57)	1077 (5.00)
Nephrouretectomy	405 (0.19)	4854 (18.7)	<0.0001	3306 (77.6)	1508 (7.00)
Solitary kidney	15 (0.01)	58 (0.22)	<0.0001	23 (0.54)	35 (0.16)

B-UC: bladder-urothelial carcinoma, NTD: New Taiwan dollar, SD: standard deviation, UC: urothelial carcinoma, UT-UC: upper urinary tract-urothelial carcinoma.

a **P** values were evaluated by chi-square test between UC patients and comparison group.

### Incidence and hazard ratios of ESRD in the different cohorts

**Figure 1:**
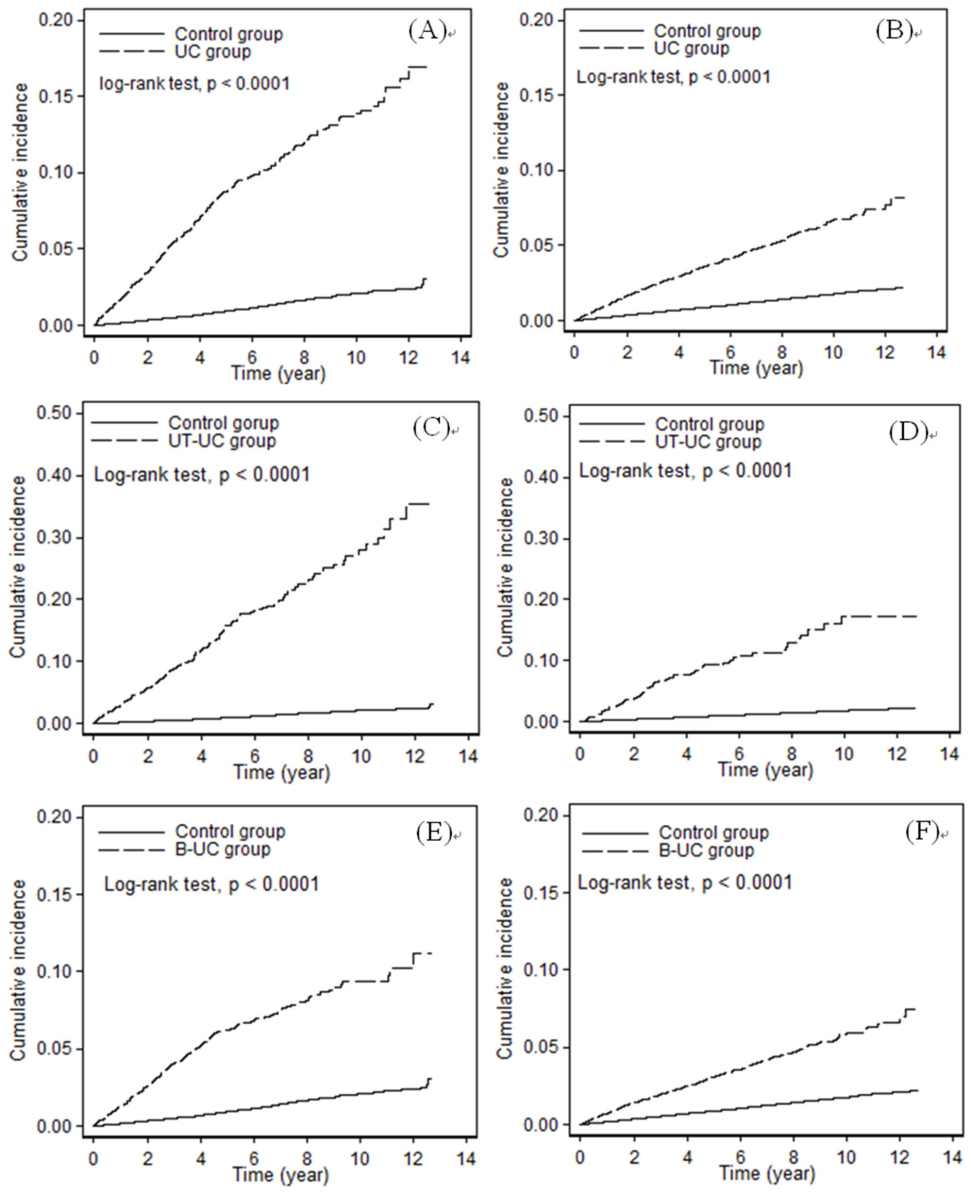
Cumulative incidence of ESRD in the control cohort and the UC cohort (A. men, B. women), the UT-UC cohort (C. men, D. women), and the B-UC cohort (E. men, F. women)

The UC, UT-UC, and B-UC groups had significantly higher ESRD cumulative incidence rates than the comparison group, and these patterns were similar for men and women (Figure 1, *p* < 0.0001 for all comparisons). In addition, women and men in the UC and B-UC cohorts had similar 3- to 6-fold increased risk for ESRD after adjusting for possible confounding factors (data not shown). Men and women in the UT-UC cohort had the greatest risk for ESRD. Overall, women had a greater risk of ESRD than men, whether in the UC, UT-UC, or B-UC cohort. Moreover, UC patients who received and did not receive nephrouretectomies had increased risks for ESRD, although the hazard ratios (HRs) were greater for those who received nephrouretectomies (Table 2). Except the analysis of stratification, we further added the factor of nephrouretectomies in the models. The significant results for increased risk of ESRD still observed. In addition, we explore the medicine use of aristolochic acids (AA) by above analyzed methods, and found the similar results for increased risk of ESRD.

**Table 2:** Multivariate Cox proportional hazard models for prediction of ESRD in different cohorts of patients who received and did not receive nephrouretectomies or AA

	N of subjects	N of ESRD	Person-years	Rate^1^	Age, sex-adjusted HR (95% CI)	Model 1^a^	Model 2
All							
Comparison Group	208136	1829	997339	1.83	1.00	1.00	1.00
UC (Total)	26017	979	98929	9.90	5.46 (5.05–5.91)***	3.87 (3.57–4.19)***	
Without Nephrouretectomy	21163	553	82887	6.67	3.72 (3.38-4.09)***	2.74 (2.48-3.01)***	2.76^b^ (2.51-3.04)***
With Nephrouretectomy	4854	426	16042	26.56	13.8 (12.4-15.4)***	8.88 (7.95-9.93)***	
Without AA	21694	771	82188	9.38	5.19 (4.77-5.64)***	3.69 (3.38-4.02)***	3.18^c^ (2.92-3.46)***
With AA	4323	208	16740	12.43	6.78 (5.87-7.82)***	4.73 (4.09-5.46)***	
UT-UC	4263	330	12515	16.37	13.5 (11.9–15.2)***	7.75 (6.84–8.78)***	
Without Nephrouretectomy	957	73	2221	32.87	16.6 (13.1-21.0)***	7.07 (5.55-9.01)***	3.20^b^ (2.65-3.86)***
With Nephrouretectomy	3306	257	10294	24.97	13.0 (11.4-14.8)***	8.25 (7.20-9.45)***	
Without AA	3326	250	9741	25.66	13.1 (11.5-15.0)***	7.75 (6.75-8.89)***	6.06^c^ (5.32-6.91)***
With AA	937	80	2774	28.84	14.6 (11.7-18.4)***	7.80 (6.20-9.82)***	
B-UC	21537	642	85750	7.49	4.18 (3.82–4.57)***	3.12 (2.84–3.42)***	
Without Nephrouretectomy	20029	475	80137	5.93	3.31 (2.99-3.66)***	2.50 (2.26-2.77)***	2.72^b^ (2.47-3.00)***
With Nephrouretectomy	1508	167	5613	29.75	15.9 (13.6-18.6)***	10.6 (8.99-12.4)***	
Without AA	18184	516	71919	7.17	4.00 (3.62-4.41)***	2.97 (2.69-3.28)***	2.63^c^ (2.39-2.90)***
With AA	3353	126	13831	9.11	5.11 (4.26-6.12)***	3.89 (3.24-4.66)***	

1 Rate per 1000 person-years

ESRD: end-stage renal disease, HR: hazard ratio.

a Multivariate model adjusted for age, sex, geographic region, occupation, monthly income, and comorbidities (diabetes, hypertension, coronary heart disease, atrial fibrillation, heart failure, chronic kidney disease, and hyperlipidemia).

b Add adjusted for nephrouretectomy in model 1.

c Add adjusted for AA in model 1.

### Association between clinical management of UC and ESRD

Among all UC patients, 8.2% received segmental ureterectomy and 26% received ureteral reimplantation (Table 3). These groups had approximately 1.3-fold (segmental ureterectomy) and 2.4-fold (ureteral reimplantation) greater risk for ESRD after control for confounding. Among UC patients, 13.7% used cisplatin and 6.1% used carboplatin, and these patients had reduced risk for ESRD; cisplatin (but not carboplatin) also had a protective effect in patients with B-UC. For UT-UC patients, those who received radical cystectomies had a 5.5-fold increased risk for ESRD compared to those who did not receive this surgery.

**Table 3:** Multivariate Cox proportional hazards model for the prediction of ESRD in all UC patients who received different clinical treatments (Yes vs. No)

	UC N (%)	Multivariate-HRs (95% CI)^a^	UT-UC N (%)	Multivariate-HRs (95% CI)^a^	B-UC N (%)	Multivariate-HRs (95% CI)^a^
Endoscopic treatment of bladder	20393 (73.4)	0.94 (0.81-1.10)	1238 (29.0)	1.00 (0.79-1.27)	19105 (88.7)	0.87 (0.67-1.13)
Radical Cystectomy	2528 (9.72)	1.17 (0.77-1.77)	69 (1.62)	4.86 (2.30-10.3)***	2442 (11.3)	0.86 (0.53-1.39)
Segmental ureterectomy	2139 (8.22)	1.29 (1.08-1.55)**	1385 (32.5)	1.06 (0.83-1.36)	742 (3.45)	1.58 (1.19-2.09)**
Endoscopic treatment of ureter	337 (1.30)	1.23 (0.81-1.86)	161 (3.78)	1.23 (0.70-2.17)	176 (0.82)	0.93 (0.50-1.76)
Ureteral reimplantation	6753 (26.0)	2.32 (2.01-2.67)***	2331 (54.7)	1.99 (1.52-2.59)***	4380 (20.3)	2.32 (1.96-2.74)***
Diversion						
Conduits	96 (0.37)	1.22 (0.45-3.29)	9 (0.21)	NA	87 (0.40)	1.93 (0.70-5.31)
Continent Cutaneous Diversions	676 (2.60)	0.90 (0.50-1.59)	27 (0.63)	0.28 (0.06-1.34)	645 (2.99)	1.07 (0.58-1.99)
Orthotopic Neobladders	1370 (5.27)	0.84 (0.52-1.38)	21 (0.49)	NA	1340 (6.22)	1.12 (0.65-1.93)
Nephrouretectomy	4854 (18.7)	1.91 (1.59-2.28)***	3306 (77.6)	0.97 (0.72-1.29)	1508 (7.00)	2.26 (1.76-2.90)***

a Multivariate model adjusted for age, sex, geographic region, occupation, monthly income, and comorbidities (diabetes, hypertension, coronary heart disease, atrial fibrillation, heart failure, chronic kidney disease, and hyperlipidemia).

NA: UT-UCC patients receiving conduits or orthotopic neobladders had no ESRD incidence.

### Joint effect of comorbidities and UC on ESRD risk

Comparisons of the UC and comparison cohorts and of the different UC subtypes with the control cohort indicated that patients with CKD, diabetes, and hypertension had significantly increased risk for ESRD (data not shown). Therefore, we calculated the multivariate-adjusted HRs for a combination of comorbidities with UC on the risk for ESRD (Table 4). The results show that UC patients with CKD had the greatest risk for ESRD in all comparisons (Table 4). There were significant joint effects of CKD with UT-UC (interaction p value = 0.0003). In addition, there were also significant joint effects of diabetes and hypertension with UC, with similar results for the UT-UC and B-UC cohorts (data not shown).

**Table 4:** Interaction between CKD, UC, and UC subtype type on the risk for ESRD

		Number, CSS/CN	Multivariate HR (95% CI)^a^	Trend p values
CKD	UC			
No	No	1644/206870	1.00	<0.0001
	Yes	804/25457	3.93 (3.61–4.29)***	
Yes	No	185/1266	16.5 (14.0–19.4)***	
	Yes	175/560	57.6 (48.9–67.8)***	
CKD	UT-UC			
No	No	1644/206870	1.00	<0.0001
	Yes	272/4106	8.99 (7.84–10.3)***	
Yes	No	185/1266	14.5 (12.3–17.1)***	
	Yes	58/157	71.5 (54.7–93.5)***	
CKD	B-UC			
No	No	1644/206870	1.00	<0.0001
	Yes	526/21137	3.01 (2.73–3.33)***	
Yes	No	185/1266	15.7 (13.3–18.4)***	
	Yes	116/400	50.6 (41.6–61.6)***	

a Multivariate model adjusted for age, sex, geographic region, occupation, monthly income, and comorbidities (diabetes, hypertension, coronary heart disease, atrial fibrillation, heart failure, chronic kidney disease, and hyperlipidemia).

## DISCUSSION

This 12-year follow-up study is the first large-scale investigation of the renal outcomes of UC patients in Taiwan, a region that has a high prevalence of UC. There were 3 major findings. First, UC is an independent risk factor for ESRD in male and female patients whether or not they receive nephrouretectomies. Second, women with UT-UC or B-UC have a greater risk for ESRD than men with either UC subtype. Third, patients with UT-UC are more likely to progress to ESRD than those with B-UC, particularly for those with CKD, diabetes, and hypertension.

Our findings confirm that UC patients have a greater risk for ESRD, and this greater risk occurred in those receiving and not receiving nephrouretectomies. Limited information is available on the prognostic impact of UC on renal survival. The total urinary arsenic level is related to CKD risk in a dose-dependent manner with adjustment for age and sex or for multiple risk factors in Taiwan, an area in which blackfoot disease is endemic [12]. The International Agency for Research on Cancer (IARC) classified arsenic as a group I carcinogen because exposure can lead to cancers of the skin, lung, and bladder [13]. Further research is necessary to determine the effect of arsenic exposure on renal survival of UC patients, and whether factors other than inorganic arsenic exposure contribute to the unusually high incidence of UT-UC and ESRD in areas of Taiwan where blackfoot disease is not endemic [14]. Cancer itself is often associated with abnormalities that affect the kidney, and cancer therapy often leads to acute and chronic kidney injury [15]. Furthermore, the marked improvements in cancer therapy have increased the number of cancer survivors, many of whom have residual kidney injury [16, 17].

UT-UC is an independent risk factor for ESRD. A few studies have also shown a significant influence of UT-UC on subsequent ESRD among UC patients [9], possibly because UT-UC may cause more severe obstructive uropathy than B-UC. Gupta et al. reported that B-UC patients with obstructive uropathy, radical cystectomy, and urinary diversion did not have increased risk of ESRD, provided they are adequately prepared before surgery by optimization of renal function [18]. After obstructive uropathy, renal tubular cell injury results from mechanical stretching, ischemia, hypoxia, or exposure to toxins such as oxygen-free radicals. In addition, renal tubular epithelial cells undergo cell death by apoptosis or necrosis, or undergo phenotypic transformation in which they acquire mesenchymal characteristics [19]. If the renal stimulus is transient, these changes can lead to remodeling or regeneration of the tubular epithelium; however, if the insult is severe or prolonged, it may activate a maladaptive response that leads to attraction to the interstitium of inflammatory cells, further damage the tubules, and renal interstitial fibrosis [19]. Our previous findings confirm that patients with CKD, but not necessarily ESRD, have a higher prevalence of UT-UC [4]. This observation is consistent with the close relationship observed here between UT-UC and obstructive uropathy. Moreover, our multivariate analysis indicated that ureteral reimplantation due to obstructive uropathy significantly increased the risk for ESRD (Table 3).

In our series, higher percent of UT-UC patients (22%) did not receive nephroureterectomy. Nephroureterectomy is the gold standard for managing UT-UC. Almost 40% of UT-UC patients receiving nephroureterectomy experienced a perioperative complications, and poor performance status conferred a four-fold greater risk of a perioperative complication [20]. However, many patients with UT-UC who require nephroureterectomy in our study are elderly, comorbid, and at risk for perioperative complications (Table 1). Thus, after survey by urologist, higher percent of UT-UC patients did not receive nephroureterectomy in Taiwan.

The results of the present study indicate that the risk for ESRD was greater in female than male patients with UC, although the reason for the greater risk in females remains unknown. Previous research indicated a greater percentage of females than males prefer alternative therapies, such as Chinese herbs that contain AA. In a Taiwan cohort of 6548 Chinese herbalists, female herbalists had a greater risk for urologic cancers than males (standardized mortality ratio: 3.10; 95% CI = 1.41–5.87) [21]. Recently, Chen et al. reported that patients with AA-induced UT-UC were more likely to be female and to have more advanced renal disease [22]. This may indicate a greater exposure to AA in Taiwanese women or that women are more sensitive to AA [23]. Our data are consistent with recent epidemiologic studies which reported that approximately one-third of the population of Taiwan consumed Chinese medicinal herbs that may contain AA [23–25]. In addition, there is a linear dose–response relationship between consumption of herbal remedies that contain AA with urinary tract cancer [24] and with ESRD [25]. Consistent with their findings, we further showed that UT-UC and B-UC independently increased the risk for ESRD in patients after consumption of AA (Table 2). These epidemiologic studies likely underestimate AA exposure because they only considered *Aristolochia*-based prescriptions written by physicians over a period of 7 years, and *Aristolochia* herbs have long been available in Taiwan from alternative sources.

In the study by Chang et al, the authors report that the use of Chinese herbs, especially if they contain AA, may contribute to the development of UC [7]. A population-based study also revealed that the use of AA-containing Chinese herbal products is associated with a dose-dependent increased risk of UC and ESRD [24, 25]. In addition, we found that even segmental ureterectomy, ureteral reimplantation and nephroureterectomy had an increased for ESRD. Therefore, the authors suggest that, in addition to AA induced UC, even segmental ureterectomy, ureteral reimplantation and nephroureterectomy, had an increased risk of ESRD.

Radical cystectomy remains the standard of care for patients with muscle invasive bladder UC. ESRD has traditionally been viewed as a potential long-term consequence of urinary diversion after radical cystectomy because it increases the risk of obstructive uropathy secondary to ureteral-enteric anastomotic stricture or disease recurrence, refiltration of previously excreted toxins that are reabsorbed by the urinary diversion, and upper tract disease recurrence resulting in surgical removal [26]. However, although there was a long-term risk of ESRD after urinary diversion, in our patients with preexisting CKD, there was no significant difference in the development of ESRD in pateints given different types of diversion. Our finding of no significant difference in ESRD risk between conduits, continent cutaneous diversions, and orthotopic neobladders is consistent with that of previous single institution retrospective studies [27]. Future prospective research and alternative measures of renal function would provide more insight into the true risk of renal deterioration after urinary diversion regardless of the type of diversion.

Satasivam et al. found that the patients who possess medical risk factors for CKD are at increased risk of developing progressive renal impairment, despite the use of kidney sparing surgery [28]. More recently, in Capitanio et al's report, the patients with clinically localized T1abN0M0 renal mass, after accounting for individual baseline characteristics, such as age, diabetes, uncontrolled hypertension, or other comorbidities, showed kidney sparing surgery independently protects against ESRD and the consequent need for dialysis relative to radical nephrectomy [29]. Nevertheless, the authors suggest that, in addition to patients with pre-existing CKD, kidney sparing surgery should be strongly considered in those patients at risk for future renal impairment as predicted by age and medical comorbidities.

This study had the limitations due to the design of the RCIP dataset. First, UC and ESRD diagnoses rely on administrative claims data from physicians or hospitals, and these may be less accurate than those made from standardized criteria. However, the diagnosis of ICD-9 code 188, 189.1, 189.2, or 585 is a serious matter, so physicians generally avoid making such diagnoses unless there is supporting pathological and laboratory reports. Moreover, information on the aggressiveness of UC was not available in the database. Thus, we were unable to measure whether UC aggressiveness was associated with ESRD. Second, this is a retrospective cohort analysis that relied on billing codes to evaluate clinical outcomes, and billing codes can be inconsistent or inaccurate. Information on clinical tests, anthropometric and laboratory examinations, and health-related behaviors of patients (such as smoking and drinking) are not available in the claims data. Thus, we were unable to assess the relationships of laboratory markers, such as hemoglobin or albumin levels, with ESRD. However, the claims data enabled us to perform a natural history study of other comorbidities that may be associated with the renal outcome of patients with UC. Although evaluating CKD stage may be difficult due to inconsistent coding, diagnosis of ESRD that requires dialysis is more clearly associated with medical complications and Medicare reimbursement. In addition, episodes of acute kidney injury during the follow-up period might have increased the accumulated incidence of ESRD in the study and comparison cohorts. However, a major strength of this study is the use of a nationwide population-based sample, because this enabled us to collect representative patients with ESRD. Further long-term follow-up studies that consider other biological and clinical information are needed to elucidate the mechanism of ESRD development in patients with UC.

This large population-based retrospective cohort study suggests that patients with UC have an increased risk for ESRD within approximately 3 to 4 years following cancer diagnosis, and that this increased risk applies to patients receiving and not receiving nephrouretectomies. These relationships should be explored further to identify the possible pathological mechanisms of renal fibrosis and to identify more specific high-risk groups. Surveillance for CKD with renal function testing and ultrasonography may be appropriate for high-risk groups. Patients with UC who also have CKD, diabetes, or hypertension may therefore benefit from early referral to a nephrologist and for long-term follow-up visits.

## MATERIALS AND METHODS

### Data sources

This retrospective cohort study used the inpatient claims database and Registry for Catastrophic Illness Patients (RCIP), which are parts of the National Health Insurance Research Databases (NHIRD) released by the Taiwan National Health Insurance Administration. This health agency established a universal single-payer insurance program on March 1, 1995, and more than 96% of Taiwan residents had coverage as of 1996. A patient can apply for a claim for a catastrophic illness based on examination of medical records, laboratory studies, and imaging studies by at least two specialists. The databases contain all inpatient claims and insurance information from 1996 to 2010. The identification of insurants was recorded before release. This study was approved by the ethical review board of the China Medical University in Taiwan.

### Study subjects

We selected 34,211 patients from the RCIP who had UC (International Classification of Diseases, 9th Revision, Clinical Modification [ICD-9-CM] 188, 189.1-189.0) from 1998 to 2010. The date of UC registry in the RCIP was considered the index date. We excluded patients younger than 25 years and those with a history of another malignancy (ICD-9-CM 140-174, 189.0, 191-208), a history of ESRD (ICD-9-CM 585), or a follow-up duration less than 3 months. Finally, all 26,017 patients with UC (UC cohort) were categorized as having UT-UC (ICD-9-CM 189.1 and 189.2, *N* = 4263) or bladder UC (B-UC, ICD-9-CM 188, *N* = 21537). The comparison cohort consisted of individuals with no history of UC before the index date; individuals in this group were randomly matched (8:1) with the UC cohort in terms of age (5 year intervals), sex, index year, and index month. The comparison cohort had the same exclusion criteria as the UC cohort.

### Outcome measures

Observed outcome was the incidence of ESRD, those with receiving long-term dialysis (>3 months) and coded as ICD-9-CM 585 in the database. The study variables included sociodemographic characteristics and comorbidities. The sociodemographic variables were sex, age, geographic region of residence (northern, central, southern, and eastern), occupation (white collar, blue collar, and others), and monthly income (based on insurance premium) as less than US$528, US$528–640, and more than US$640 (corresponding to less than NTD15840, NTD15841-20100, and more than NTD20100). The comorbidities were diabetes (ICD-9-CM 250), hypertension (ICD-9-CM 401-405), coronary heart disease (ICD-9-CM 410-414), atrial fibrillation (ICD-9-CM 427.31), heart failure (ICD-9-CM 428), chronic kidney disease (ICD-9-CM 585), hyperlipidemia (ICD-9-CM 272), nephrouretectomy (ICD-9-CM operation code 55.51), and solitary kidney (ICD-9-CM 753.0). In Taiwan, CKD was diagnosed and staged according to the K/DOQI clinical practice guideline [30]. All Taiwanese patients who develop ESRD and require long-term dialysis can apply for catastrophic illness registration cards from the NHI Administration and have no co-payments for health care. The analysis also considered endoscopic treatment of the bladder (78008C, 78049C), radical cystectomy (78011B, 78039B, 78040B, 78012B, 78041B, 78043B, 78042B, 78013B, 78044B, 78014B, 78045B, 78046B), segmental ureterectomy (77003B, 77004B, 77005B, 77006B, 77007B), endoscopic treatment of the ureter (77034B), ureteral reimplantation (50010C, 50019C), conduit diversion (77013B, 77014B, 77015B, 77016B, 77017B), continent cutaneous diversion (77018B, 77019B, 77020B, 77022B), orthotopic neobladder (78012B, 78014B, 78041B, 78045B), and use of AA (A004143, A016919, A019285, A035747, A037982, A044152, A045727, A046081, A046918, A055627, A054560, A002821, A017465, A021356, A034838, A039470, A046913, A047113, A055457, A055646, A006926, A016115, A032033, A032659, A034698, A035728, A039840, A040474, A040904, A043424, A056512, A056413, A034629, A043131, A034629, A034417, A038536, A037415, A035138, A040480, A036897, A031705, A042567, A042787, A039869, A036566, A037455, A034500, A036121, A034497, A032078, A037818, A035116, A037647, A045841, A039113, A043054, A043556, A035907, A042689, A038343, A037459, A035120, A032546, A035725, A032241, A038715, A037113, A036124, A037820, A036989, A037102, A038232). All comorbidities were recorded at admission.

### Statistical analysis

Person-years were defined as the interval from the index date to the date of ESRD diagnosis, loss to follow-up, death, or December 31, 2010. We estimated the overall cumulative incidence and incidence by sex, UC, and UC subtype from 1998 to 2010. A chi-square test was used to assess the significance of differences in sociodemographic factors and comorbidities in the comparison and UC cohorts. Kaplan–Meier analysis was employed to plot cumulative incidence curves, and the log-rank test was used to test the significance of differences in these curves between the two groups by sex. Cox proportional hazard regression analysis was used to estimate the risk of ESRD associated with UC and UC subtype. All data analyses were conducted using the SAS 9.3 statistical package for Microsoft Windows (SAS Institute, Cary, NC, USA), and the significance level was set at 0.05, using a two-sided test.
